# Central and Midperipheral Corneal Thickness Measured with Scheimpflug Imaging and Optical Coherence Tomography

**DOI:** 10.1371/journal.pone.0098316

**Published:** 2014-05-22

**Authors:** Jinhai Huang, Xixia Ding, Giacomo Savini, Zhengxuan Jiang, Chao Pan, Yanjun Hua, Fan Wu, Yifan Feng, Ye Yu, Qinmei Wang

**Affiliations:** 1 School of Optometry and Ophthalmology and Eye Hospital, Wenzhou Medical University, Wenzhou, Zhejiang, China; 2 Key Laboratory of Vision Science, Ministry of Health P.R. China, Wenzhou, Zhejiang, China; 3 Studio Oculistico d'Azeglio, Bologna, Italy; 4 Department of Ophthalmology, The Second Affiliated Hospital of Anhui Medical University, Hefei, Anhui, China; University of Melbourne, Australia

## Abstract

**Purpose:**

To compare corneal thickness measurements using Pentacam (Oculus, Germany), Sirius (CSO, Italy), Galilei (Ziemer, Switzerland), and RTVue-100 OCT (Optovue Inc., USA).

**Methods:**

Sixty-six eyes of 66 healthy volunteers were enrolled. Three consecutive measurements were performed with each device. The mean value of the three measurements was used for subsequent analysis. Central corneal thickness (CCT), thinnest corneal thickness (TCT), and midperipheral corneal thickness (MPCT; measured at superior, inferior, nasal, and temporal locations with a distance of 1 mm (CT_2mm_) or 2.5 mm (CT_5mm_) from the corneal apex) were analyzed. Differences and agreement between measurements were assessed using the repeated-measures analysis of variance (ANOVA) and Bland-Altman analyses, respectively.

**Results:**

Statistically significant differences (p<0.001) among the four devices were revealed in CCT, TCT and CT_2mm_measurements. The CCT, TCT, and CT_2mm_ values were ranked from the thickest to the thinnest as follows: Galilei>Sirius>Pentacam>RTVue OCT. For these measurements, agreement between measurements by Sirius and Pentacam was good, whereas Galilei overestimated and RTVue underestimated corneal thickness compared to Sirius and Pentacam. As regards CT_5mm_ measurements, Pentacam provided the largest values, whereas RTVue OCT yielded the smallest values. Agreement of the CT_5mm_ measurements was good between the Pentacam, Sirius, moderate between Galilei and the other two Scheimpflug systems, and poor between the RTVue OCT and the remaining devices.

**Conclusions:**

The Pentacam and Sirius can be used interchangeably for CCT measurements, while the Galilei and RTVue systematically over- and underestimate CCT, respectively. The three Scheimpflug cameras, but not the RTVue, may be used interchangeably for MPCT measurements.

## Introduction

Corneal thickness measurement is crucial in many clinical and research applications [1]. The determination of central corneal thickness (CCT) is important for the planning of refractive surgery, diagnosis of glaucoma, and monitoring of corneal edema [2]–[7]. In addition, the midperipheral corneal thickness (MPCT) assessment is also essential for diagnosis and follow-up of keratoconus and for corneal surgeries such as corneal cross-linking, radial keratotomy, and intrastromal ring placement [8]–[10].

Ultrasonic pachymetry (USP) is the gold-standard technique for measuring corneal thickness. However, it requires contact with the cornea and topical anesthesia, which may cause patient discomfort and a decrease in reliability. The reliability can also be limited by the operator's skill to manually place the USP probe as perpendicularly as possible to the center of the cornea [11]–[13]. To overcome the disadvantages of USP, many noncontact instruments have been developed. Among these, the Scheimpflug imaging systems and Fourier-domain optical coherence tomography (FD-OCT) have attracted substantial attention. The repeatability and reproducibility of corneal thickness measurements have been reported to be good with the use of these instruments [13]–[16], and particularly, the reliability of CCT and thinnest corneal thickness (TCT) measurements has been shown to be higher than the peripheral corneal thickness measurements [16], [17].

Previous studies have compared CCT and/or thinnest corneal thickness (TCT) measurements obtained by some of these instruments, such as between a single rotating Scheimpflug camera, the Pentacam (Oculus, Wetzlar, Germany) and a single rotating Scheimpflug camera combined with a Placido disc corneal topographer, the Sirius (Costruzione Strumenti Oftalmici, Florence, Italy) [18], [19]; between the Pentacam and a dual Scheimpflug camera combined with a Placido disc corneal topographer, the Galilei (Ziemer, Port, Switzerland) [14]; and between the Pentacam and the RTVue-100 FD-OCT (Optovue Inc., Freemont, CA, USA) [13], [20]–[22]. Other studies have assessed the agreement between MPCT measurements [9], [22]. To our knowledge, however, no previous study has compared CCT, TCT, and MPCT measurements obtained by the four above-mentioned devices at the same time and under the same conditions. It is not known whether the values obtained with these instruments are comparable and whether these instruments can be used interchangeably in a clinical setting.

In the current prospective study, we aimed to comprehensively assess the extent of agreement between the CCT, TCT, and MPCT measurements obtained by the Pentacam, the Sirius, the Galilei, and the RTVue-100 OCT systems.

## Subjects and Methods

### Ethics Statement

The study was conducted at the Eye Hospital of Wenzhou Medical University. The research was performed in accordance with the principles stated in the Declaration of Helsinki and was approved by the Office of Research Ethical Committee, Wenzhou Medical University. All participants provided written informed consent after the nature of the study had been explained to them.

### Subjects

This prospective study enrolled 66 right eyes of healthy volunteers (33 men, 33 women). The mean age ± standard deviation (SD) was 35.39±10.06 years (range: 18 to 55 years). The entire research procedure was performed at the Eye Hospital of Wenzhou Medical University. This study adhered to the Declaration of Helsinki principles and was approved by the Office of Research Ethics, Wenzhou Medical University. Written informed consent was obtained from each subject.

Before inclusion in the study, all patients underwent a complete ophthalmic examination including refraction, slit-lamp microscopy, ophthalmoscopy, noncontact tonometry, and corneal topography (ALLEGRO Topolyzer™; WaveLight Technologie AG, Erlangen, Germany). The exclusion criteria included a history of contact lens use (rigid contact lens within 4 weeks and soft contact lens within 2 weeks); corrected vision less than 20/20; intraocular pressure of more than 21 mmHg, as measured by noncontact tonometry; high corneal astigmatism (more than 2 D); active ocular pathology; and a history of eye surgery.

### Instruments

The Pentacam system uses a rotating Scheimpflug camera (180 degrees) and a monochromatic slit-light source (blue light-emitting diode [LED], at 475 nm), which provides a 3-dimensional scan of the anterior segment of the eye. During the scanning, the camera and light source rotate together around the optical axis of the eye. The rotating camera captures 25 slit images are obtained in less than 2 seconds. Simultaneously, the eye movements are detected using a second pupil camera and are automatically corrected during the calculation process.

Sirius, a relatively new imaging system, is based on the combination of a rotating Scheimpflug camera and a small-angle Placido disk topographer with 22 rings. A full scan acquires a series of 25 Scheimpflug images (meridians) and one Placido top-view image. Ring edges and height slope are obtained from the Placido image, and the curvature data are obtained by the arc-step method with conic curves. The profiles of anterior cornea, posterior cornea, anterior lens, and iris are provided by the Scheimpflug images. The Placido image and the Scheimpflug images are merged using a proprietary method to finally determine the data of the anterior corneal surface.

The Galilei imaging system combines a dual Scheimpflug camera and a Placido disk to measure both anterior and posterior corneal surfaces. A full scan can be performed within 1 or 2 seconds. The corneal data are simultaneously obtained by the Placido and Scheimpflug systems, and the anterior corneal measurements are determined using a proprietary method that merges the two types of data. The dual camera captures two Scheimpflug slit images from opposite sides of the illuminated slit, and the data are averaged. Meanwhile, the dual camera simultaneously tracks decentration due to eye movements.

The RTVue-100 OCT imaging system is based on the FD-OCT technology. With 5 µm of depth resolution in tissue, it provides high-magnification imaging of the cornea within 0.04 seconds. A super-luminescence diode is used as a low-coherence light source, which emits light with a 50-nm bandwidth centered at 830 nm. A cornea-anterior module long (CAM-L) lens adapter with a low magnification is mounted on the probe to focus the OCT beam onto the cornea. The corneal pachymetry protocol that is used to perform the scanning acquires eight evenly spaced 6-mm radial lines oriented at 22.5° from one another, consisting of 1024 A-scans per line in 0.31 s. In the present study, all FD-OCT measurements were performed using the apex-centered mode. A bright and vertical flare line was placed at the center of the real-time OCT image by adjusting the position of the OCT system.

### Measurement Technique

Corneal thickness measurements on each subject were performed by a single well-trained operator (P.C.) who was experienced in using all four instruments. The order in which the instruments were used was randomized. According to the manufacturer's guidelines, all eye measurements were performed without dilation in a dim room between 10 AM and 5 PM to minimize diurnal changes in corneal shape and thickness [23]. After acquiring each scan and checking that the “examination quality specification” was OK, the operator saved the examination but did not open it and read the measured values.

Subjects were positioned with a headrest and asked to fixate on an internal fixation within each device. They were instructed to blink completely just before each scanning to allow an optically smooth tear film to spread over the cornea. After each scan acquisition, the patient was asked to sit back, and the device was realigned for the next scan. Only scans with an “examination quality specification” of “OK” were retained for analysis. About 5% of the acquired scans were deleted, and the scans were deleted and repeated due to insufficient quality.

For each subject, three consecutive and standard measurements were performed using each system. The three measurements were averaged to determine the mean value of corneal thickness obtained by each device. The difference between the mean values was used to assess the agreement of corneal thickness measurements among the four instruments.

In the present study, corneal thickness measurements obtained by each system included CCT, TCT, and MPCT. CCT at the corneal apex point was measured using each device. TCT was obtained at the thinnest point of the cornea. MPCT was measured at the superior, inferior, nasal, and temporal locations with a distance of 1 mm and 2.5 mm from the corneal apex (i.e., with a diameter of 2 mm and 5 mm, respectively). Therefore, eight categories of MPCT were chosen for analysis that included four directions and two distances. Those categories using a diameter of 2 mm were defined as CT_2mm_ (CT_superior-2mm_, CT_inferior-2mm_, CT_nasal-2mm_, and CT_temporal-2mm_), and those using a diameter of 5 mm were defined as CT_5mm_ (CT_superior-5mm_, CT_inferior-5mm_, CT_nasal-5mm_, and CT_temporal-5mm_).

### Statistical analysis

Statistical analysis was performed using SPSS software for Windows version 17 (SPSS Inc., Chicago, IL, U.S.) and Microsoft Office Excel. A P value of less than 0.05 was considered to be statistically significant. The distributions of the datasets were checked for normality using Kolmogorov–Smirnov tests. The results indicated that the data were normally distributed (*p*>0.05).

For multiple comparisons between the corneal thickness measurements, the repeated-measures analysis of variance (ANOVA) with Bonferroni post hoc comparison procedure was used. Agreement between the 4 devices was assessed using Bland-Altman plot analysis. In this graphical method, the differences between the 2 devices are plotted against the averages of the 2 devices. The difference between the two devices is displayed in the Y axis, while the mean value in the X axis. The 95% limits of agreement (LoA) were calculated as the average difference in measurements from the two devices ±1.96 SD [24].

## Results

### Agreement between CCT, TCT, and CT_2mm_ measurements obtained by the four systems

The mean value of each measured parameter is reported in Table 1. The RTVue OCT provided the thinnest values for all locations, whereas the Galilei imaging system provided the thickest values for central and midperipheral corneal measurements at 2 mm. Consequently, the lowest mean differences for the CCT and TCT measurements fell between the mean measurements obtained by the Sirius and Pentacam systems, and the mean values of the Pentacam were approximately 3 µm thinner than those of the Sirius system. Repeated-measures ANOVA revealed statistically significant differences for all parameters measured by all four instruments (*P*<0.001). According to the Bonferroni post-test (Tables 2–4 and Tables S1–S3), only the mean difference between CT_inferior-2mm_ measurements obtained by the Pentacam and Sirius imaging systems was not statistically significant (P = 0.129; Tables 2–4 and Tables S1–S3).

**Table 1:** Central corneal thickness, thinnest corneal thickness and midperipheral (2 and 5 mm diameter) corneal thickness in the superior, inferior, nasal, and temporal quadrants with the four investigated devices.

Parameter	Mean (µm) ± Standard Deviation				*P* Value
	Pentacam	Sirius	Galilei	RTVue	
Center	538.8±26.5	542.1±27.1	548.1±26.4	532.8±26.2	<0.001
Thinnest	535.8±26.4	539.2±27.1	545.6±26.4	528.1±26.5	<0.001
Superior 2 mm	555.4±27.3	559.3±27.8	563.1±27.0	542.4±26.6	<0.001
Inferior 2 mm	542.5±26.8	544.0±27.2	551.3±26.4	533.4±26.8	<0.001
Nasal 2 mm	549.4±26.8	552.8±27.4	558.7±26.5	540.0±26.8	<0.001
Temporal 2 mm	540.1±26.7	545.4±27.5	550.7±27.1	533.2±26.3	<0.001
Superior 5 mm	626.2±30.9	625.8±30.3	619.1±30.0	591.7±29.2	<0.001
Inferior 5 mm	592.1±28.3	587.0±29.0	588.0±27.1	562.4±28.5	<0.001
Nasal 5 mm	605.5±28.4	600.9±28.6	602.9±27.5	575.5±28.1	<0.001
Temporal 5 mm	585.4±28.3	582.4±29.2	580.0±28.9	554.3±27.6	<0.001

**Table 2:** Mean difference of central corneal thickness, corresponding results of Bonferroni post hoc comparison and 95% limits of agreement (LoA) among the 4 investigated devices.

Device Pairings	Mean Difference (µm) ± SD	*P* Value	95% LoA (µm)
Pentacam - Sirius	−3.3±5.2	<0.001	−13.6 to 6.9
Pentacam - Galilei	−9.3±3.7	<0.001	−16.6 to −2.0
Pentacam - RTVue	6.0±4.8	<0.001	−3.4 to 15.4
Sirius - Galilei	−6.0±4.0	<0.001	−13.8 to 1.9
Sirius - RTVue	9.3±5.6	<0.001	−1.6 to 20.3
Galilei - RTVue	15.3±4.1	<0.001	7.3 to 23.3

SD =  Standard deviation.

**Table 3:** Mean difference of thinnest corneal thickness, corresponding results of Bonferroni post hoc comparison and 95% limits of agreement (LoA) among the 4 investigated devices.

Device Pairings	Mean Difference (µm) ± SD	*P* Value	95% LoA (µm)
Pentacam - Sirius	−3.4±5.2	<0.001	−13.5 to 6.7
Pentacam - Galilei	−9.8±3.7	<0.001	−17.0 to −2.7
Pentacam - RTVue	7.7±5.2	<0.001	−2.5 to 18.0
Sirius - Galilei	−6.4±3.8	<0.001	−13.9 to 1.1
Sirius - RTVue	11.1±6.0	<0.001	−0.7 to 22.9
Galilei - RTVue	17.6±4.1	<0.001	9.6 to 25.5

SD =  Standard deviation.

**Table 4:** Mean difference of superior 2% limits of agreement (LoA) among the 4 investigated devices.

Device Pairings	Mean Difference (µm) ± SD	*P* Value	95% LoA (µm)
Pentacam - Sirius	−4.0±5.5	<0.001	−14.7 to 6.8
Pentacam - Galilei	−7.8±4.5	<0.001	−16.7 to 1.1
Pentacam - RTVue	12.9±5.0	<0.001	3.2 to 22.6
Sirius - Galilei	−3.8±4.7	<0.001	−13.0 to 5.4
Sirius - RTVue	16.9±5.6	<0.001	5.9 to 28.0
Galilei - RTVue	20.7±4.8	<0.001	11.3 to 30.1

SD =  Standard deviation.

The 95% LoAs are reported in Tables 2–4, Tables S1–S3, and Figures S1–S6. According to these data, the Sirius overestimated the CCT, TCT, and CT_2mm_ when compared to the Pentacam. In addition, Bland-Altman plots confirmed that the Galilei overestimated and that the RTVue system underestimated the CCT, TCT, and CT_2mm_ in comparison to the other devices.

### Agreement between CT_5mm_ measurements obtained by all the four systems

The mean value of each measured parameter is reported in Table 1. In the case of CT_5mm_ measurements, the RTVue OCT system provided the thinnest values for all locations measured. The Pentacam system provided the thickest measurements in all categories (Tables 5 and Tables S4–S6). The repeated-measures ANOVA revealed statistically significant differences for corneal thickness measurements obtained at all locations by the four devices (*P*<0.001). The Bonferroni post-hoc test demonstrated a statistically significant difference between each pair of instruments, except the following ones: Pentacam vs. Sirius for CT_superior- 5mm_, Pentacam vs. Galilei for CT_nasal -5mm_, and Sirius vs. Galilei for CT_nasal -5mm_. However, no mean differences in measurements obtained by the three Scheimpflug cameras were clinically significant, whereas only the FD-OCT-based instrument provided thinner CT_5mm_ measurements in all quadrants. Accordingly, the Bland-Altman plots and 95% LoAs revealed good agreement between the CT_5mm_ measurements obtained by the Pentacam, Sirius, and Galilei systems. On the contrary, the 95% LoAs between the RTVue OCT measurements and each of the other three Scheimpflug measurements showed a broad range, which implied poor agreement between their results (Tables 5 and Tables S4–S6 and Figures S7–S10).

**Table 5:** Mean difference of superior 5% limits of agreement (LoA) among the 4 investigated devices.

Device Pairings	Mean Difference (µm) ± SD	*P* Value	95% LoA (µm)
Pentacam - Sirius	0.4±6.1	1.000	−11.6 to 12.3
Pentacam - Galilei	7.1±7.1	<0.001	−6.9 to 21.0
Pentacam - RTVue	34.4±10.6	<0.001	13.6 to 55.3
Sirius - Galilei	6.7±7.1	<0.001	−7.2 to 20.7
Sirius - RTVue	34.1±9.7	<0.001	15.0 to 53.1
Galilei - RTVue	27.4±10.6	<0.001	6.5 to 48.2

SD =  Standard deviation.

## Discussion

Many noncontact imaging devices have been introduced in clinical practice and in research settings for the measurement of corneal thickness. Thus, it is crucial to compare the corneal measurements obtained by using the recently introduced imaging systems. To our knowledge, this is the first controlled study to comprehensively evaluate the extent of agreement between the CCT, TCT, and MPCT measurements obtained using the four systems, Pentacam, Sirius, Galilei, and RTVue OCT, simultaneously. Our data suggests that good agreement in the corneal measurements was not always achieved by these imaging systems.

In case of CCT and TCT measurements, the values obtained by the Pentacam and Sirius systems were comparable and, on average, their difference may be considered not clinically significant (i.e., they should not influence clinical decisions such as the possibility to perform or not LASIK). On the contrary, the Galilei system provided thicker measurements, and the RTVue-100 OCT yielded thinner measurements. The underestimation of corneal thickness by the former and the overestimation by the latter cannot be overlooked. Given the remarkable importance of TCT in planning corneal refractive surgery using an excimer laser, we suggest that the RTVue-100 OCT and Galilei systems should not be used interchangeably.

Our results are in good agreement with results of previous studies in that several authors have already reported that the RTVue-100 OCT imaging system underestimated the value of CCT compared to that provided by the Pentacam system [19]–[21]. In accordance with our data, previous studies also found that the Pentacam system underestimated the CCT value as compared to the Galilei system [14], and the Sirius system provided slightly higher TCT measurements compared to that obtained from the Pentacam system [18]. Different results, however, have been reported by other studies. Bedei et al. [19] found that the mean CCT values yielded by Pentacam were higher (by approximately 20 µm) than those of Sirius in 30 healthy eyes of 30 subjects. Nam et al. [13] showed that the CCT values obtained by the RTVue-100 system were thicker than that obtained by the Pentacam system, whereas the TCT values yielded by the RTVue-100 system were equivalent to that provided by of the Pentacam system for normal eyes. These discrepancies may be explained by a very small sample size and inter-device differences of the same model of the imaging system.

As far as agreement in concerned, our study showed similar results to those reported by Chen et al. [22] when comparing the Pentacam and the RTVue-100 systems (95% LoA: from −0.7 µm to +22.5 µm). In contrast, our results showed better agreement between instruments with respect to previous studies. Both Savini et al. [18] and Hosseini et al. [14] reported wider 95% LoAs than those determined in our study, when comparing TCT measurements between the Sirius and the Pentacam systems (from −34.6 µm to +48.9 µm and from −10.4 µm to +27.3 µm, respectively). As a possible explanation for the results reported by Savini et al. [18], we speculate that the mean age of their study population was considerably higher (57.9±21.2 years) than that of our study population, and older patients might show poorer cooperation and worse fixation. The 95% LoAs (from −12.2 µm to +16.5 µm) were also reported between corneal measurements obtained from the Pentacam and RTVue-100 systems by Nam et al. [13] in similarly young subjects.

In addition to the CCT, TCT, and CT_2mm_ measurements, we also evaluated the agreement between CT_5mm_ measurements performed with the four systems. To our knowledge, this is the first study to assess the agreement of MPCT measurements obtained by these instruments. Previous studies comparing MPCT measurements obtained by the Pentacam and a time-domain OCT system (Visante, Carl Zeiss Meditec, Dublin, CA) showed that Scheimpflug imaging provided thicker measurements [9]. Similar results have been reported by Milla et al. [25] in a comparison of the Sirius and the Visante OCT systems. In the present study, the Pentacam system provided the largest values for CT_5mm_, whereas the RTVue-100 OCT system yielded the smallest values (Tables 5 and tables S4–S6). Bland-Altman plots revealed that there was a good agreement between the results obtained by the 3 Scheimpflug cameras, although to a lesser extent compared to central measurements, and a poor agreement between the results of the FD-OCT system and each of the Scheimpflug cameras. Agreement between the RTVue FD-OCT measurements and the 3 Scheimpflug camera measurements was even lower than for central measurements. The lower level of agreement for the 5-mm measurements (with respect to central and 2-mm measurements) may be explained by the larger variability of peripheral corneal thickness measurements, as shown by previous studies [17], [26]–[28]. We also believe that agreement of the three Scheimpflug cameras for CT_5mm_ is high enough to consider the differences not clinically significant. The 95% LoA, in fact, are close to the reported diurnal fluctuation of CCT (18 microns) and the repeatability of CCT as measured by noncontact specular microscopy and confocal microscopy [29]–[31].

There are some reasons for the statistically significant differences in corneal thickness measured using these devices. Firstly, because corneal thickness is theoretically defined as the radial distance between two concentric spheres [32], and both corneal surfaces are neither spheric nor concentric, different values may be obtained if the instruments take measurements along different axes. Discrepancies among instruments may also be related to different algorithms used by the manufacturers for corneal thickness calculation. Secondly, differences in the time needed for scanning the cornea and in the intensity of the fixation light may influence the fixation stability of the patient and subsequent measurements. The three Scheimpflug systems all need less than 2 seconds for each scanning (although measurements require a longer time with the Pentacam), while the RTVue-100 OCT imaging system only takes 0.31second. It may also be possible that different reference systems for calibration and the presence of a second Scheimpflug camera (in the case of the Galilei system) may play a role. Finally, a possible role of the tear film thickness cannot be excluded as it may affect differently Scheimpflug and OCT images, but further studies are needed to assess its influence on corneal thickness measurements by different technologies [25].

In the present study, one potential limitation is that all measurements were obtained only from healthy eyes with normal corneas. This population was chosen, because the aim of our study was to evaluate the agreement of corneal thickness measurements using the Pentacam, Sirius, Galilei, and RTVue-100 OCT imaging systems in normal subjects with good vision and fixation. Further studies are needed to assess the agreement of measurements in patients with unusual corneas, such as keratoconus or post-refractive surgery eyes. In addition, we did not include USP measurements; however, our previous studies have compared the difference and agreement of the Pentacam and RTVue OCT measurements with USP [20], [22]. Additionally, several studies have assessed the degrees of agreement in CCT measurements obtained by the Galilei and USP systems in different clinical settings [14], [33], [34]. Further research is needed to corroborate which device is closer to USP in measuring corneal thickness under the same clinical conditions, and some conversion equations can be proposed to compare CCT measurements obtained by these different noncontact instruments with USP.

In conclusion, good agreement was found between the CCT, TCT, and CT_2mm_ measurements using two of the four systems, i.e., the Pentacam and Sirius systems, which implied that they can be used interchangeably for measurements at those corneal locations. In contrast, the clinicians should be cautious about the central measurement results obtained with the Galilei and RTVue-100 OCT systems and take into consideration the possible thickness overestimation by the former and underestimation by the latter. The results of the Pentacam, Sirius, and Galilei systems showed good agreement for the measurement of CT_5mm_, and these instruments can be used interchangeably. However, poor agreement was found between corneal measurements obtained by the RTVue-100 OCT and the 3 Scheimpflug imaging systems, indicating that RTVue-100 OCT cannot be used interchangeably with other three devices.

## Supporting Information

Figure S1 **Bland-Altman plots of agreement in the central corneal thickness (CCT) measurement among Pentacam, Sirius, Galilei, and RTVue OCT.** The solid line indicates the mean difference (bias). The upper and lower lines represent the 95% LoA.
(TIF)

Figure S2 **Bland-Altman plots of agreement in the thinnest corneal thickness (TCT) measurement among Pentacam, Sirius, Galilei, and RTVue OCT.** The solid line indicates the mean difference (bias). The upper and lower lines represent the 95% LoA.
(TIF)

Figure S3 **Bland-Altman plots of agreement in corneal thickness measurement of the superior location with a distant of 1 mm from the corneal apex (CT_superior-2mm_) among Pentacam, Sirius, Galilei, and RTVue OCT.** The solid line indicates the mean difference (bias). The upper and lower lines represent the 95% LoA.
(TIF)

Figure S4 **Bland-Altman plots of agreement in corneal thickness measurement of the inferior location with a distant of 1 mm from the corneal apex (CT_inferior-2mm_) among Pentacam, Sirius, Galilei, and RTVue OCT.** The solid line indicates the mean difference (bias). The upper and lower lines represent the 95% LoA.
(TIF)

Figure S5 **Bland-Altman plots of agreement in corneal thickness measurement of the nasal location with a distant of 1 mm from the corneal apex (CT_nasal-2mm_) among Pentacam, Sirius, Galilei, and RTVue OCT.** The solid line indicates the mean difference (bias). The upper and lower lines represent the 95% LoA.
(TIF)

Figure S6 **Bland-Altman plots of agreement in corneal thickness measurement of the temporal location with a distant of 1 mm from the corneal apex (CT_temporal-2mm_) among Pentacam, Sirius, Galilei, and RTVue OCT.** The solid line indicates the mean difference (bias). The upper and lower lines represent the 95% LoA.
(TIF)

Figure S7 **Bland-Altman plots of agreement in corneal thickness measurement of the superior location with a distant of 2.5 mm from the corneal apex (CT_superior-5mm_) among Pentacam, Sirius, Galilei, and RTVue OCT.** The solid line indicates the mean difference (bias). The upper and lower lines represent the 95% LoA.
(TIF)

Figure S8 **Bland-Altman plots of agreement in corneal thickness measurement of the inferior location with a distant of 2.5 mm from the corneal apex (CT_inferior-5mm_) among Pentacam, Sirius, Galilei, and RTVue OCT.** The solid line indicates the mean difference (bias). The upper and lower lines represent the 95% LoA.
(TIF)

Figure S9 **Bland-Altman plots of agreement in corneal thickness measurement of the nasal location with a distant of 2.5 mm from the corneal apex (CT_nasal-5mm_) among Pentacam, Sirius, Galilei, and RTVue OCT.** The solid line indicates the mean difference (bias). The upper and lower lines represent the 95% LoA.
(TIF)

Figure S10 **Bland-Altman plots of agreement in corneal thickness measurement of the temporal location with a distant of 2.5 mm from the corneal apex (CT_temporal-5mm_) among Pentacam, Sirius, Galilei, and RTVue OCT. The solid line indicates the mean difference (bias).** The upper and lower lines represent the 95% LoA.
(TIF)

Table S1 **Mean difference of inferior 2 mm corneal thickness, corresponding results of Bonferroni post hoc comparison and 95% limits of agreement (LoA) among the 4 investigated devices.**
(DOCX)

Table S2 **Mean difference of nasal 2 mm corneal thickness, corresponding results of Bonferroni post hoc comparison and 95% limits of agreement (LoA) among the 4 investigated devices.**
(DOCX)

Table S3 **Mean difference of temporal 2 mm corneal thickness, corresponding results of Bonferroni post hoc comparison and 95% limits of agreement (LoA) among the 4 investigated devices.**
(DOCX)

Table S4 **Mean difference of inferior 5 mm corneal thickness, corresponding results of Bonferroni post hoc comparison and 95% limits of agreement (LoA) among the 4 investigated devices.**
(DOCX)

Table S5 **Mean difference of nasal 5 mm corneal thickness, corresponding results of Bonferroni post hoc comparison and 95% limits of agreement (LoA) among the 4 investigated devices.**
(DOCX)

Table S6 **Mean difference of temporal 5 mm corneal thickness, corresponding results of Bonferroni post hoc comparison and 95% limits of agreement (LoA) among the 4 investigated devices.**
(DOCX)
