# Supplementary figures and images for: Central and Midperipheral Corneal Thickness Measured with Scheimpflug Imaging and Optical Coherence Tomography
Source: PLoS One. 2014 May 22;9(5):e98316. doi: 10.1371/journal.pone.0098316 (PMC4031212; doi:10.1371/journal.pone.0098316)

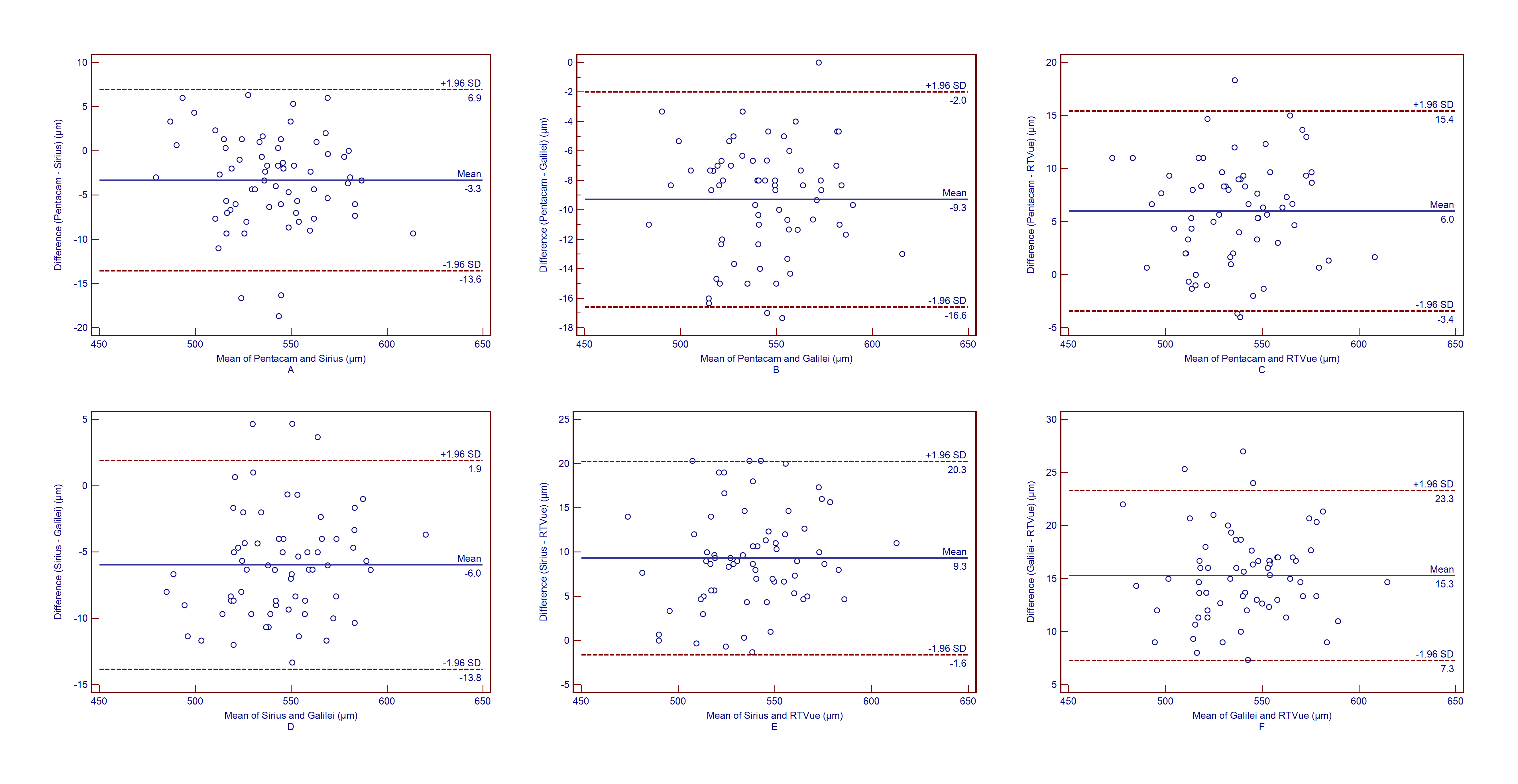

Supplement: Figure S1 — Bland-Altman plots of agreement in the central corneal thickness (CCT) measurement among Pentacam, Sirius, Galilei, and RTVue OCT. The solid line indicates the mean difference (bias). The upper and lower lines represent the 95% LoA. (TIF) [file pone.0098316.s001.tif]

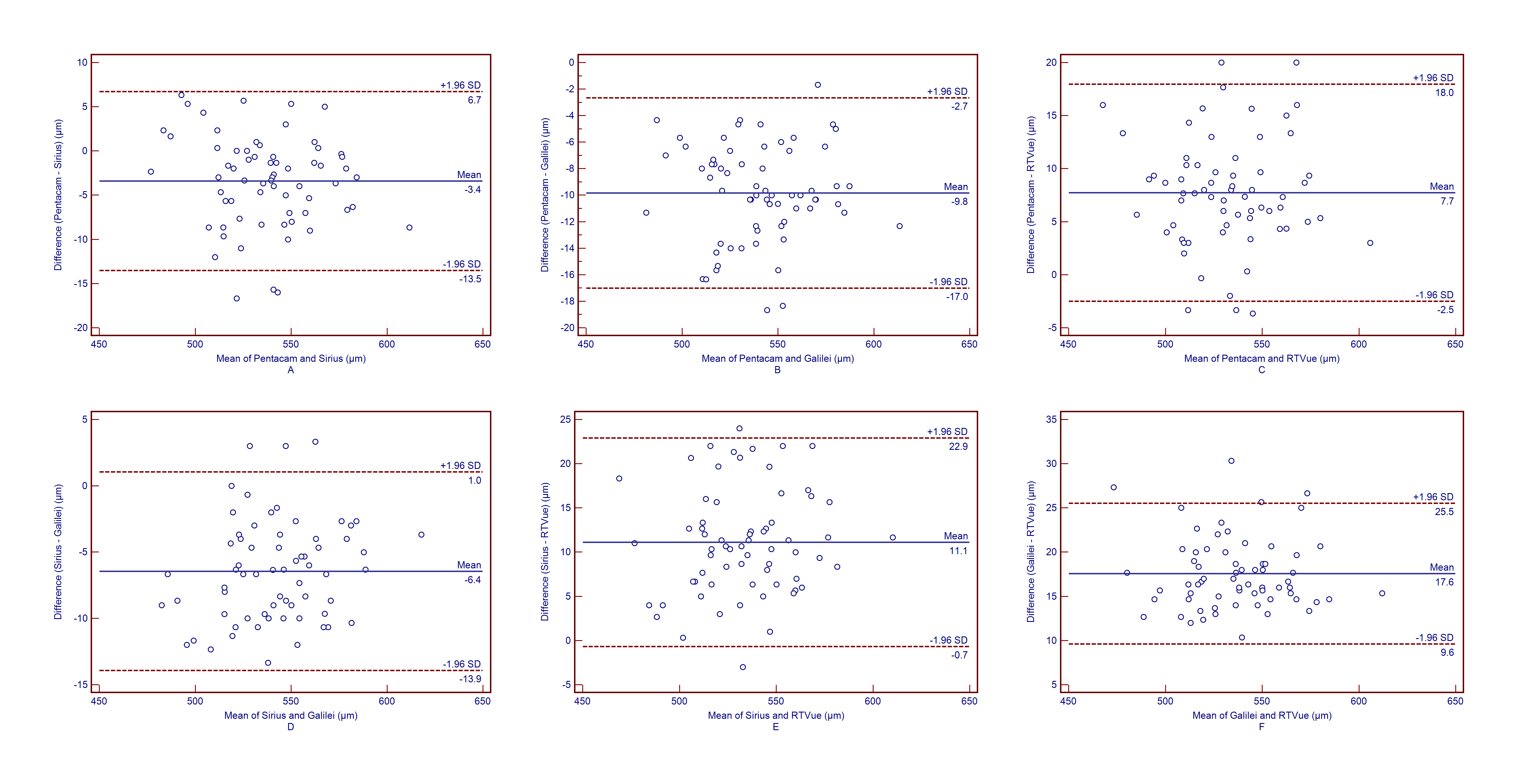

Supplement: Figure S2 — Bland-Altman plots of agreement in the thinnest corneal thickness (TCT) measurement among Pentacam, Sirius, Galilei, and RTVue OCT. The solid line indicates the mean difference (bias). The upper and lower lines represent the 95% LoA. (TIF) [file pone.0098316.s002.tif]

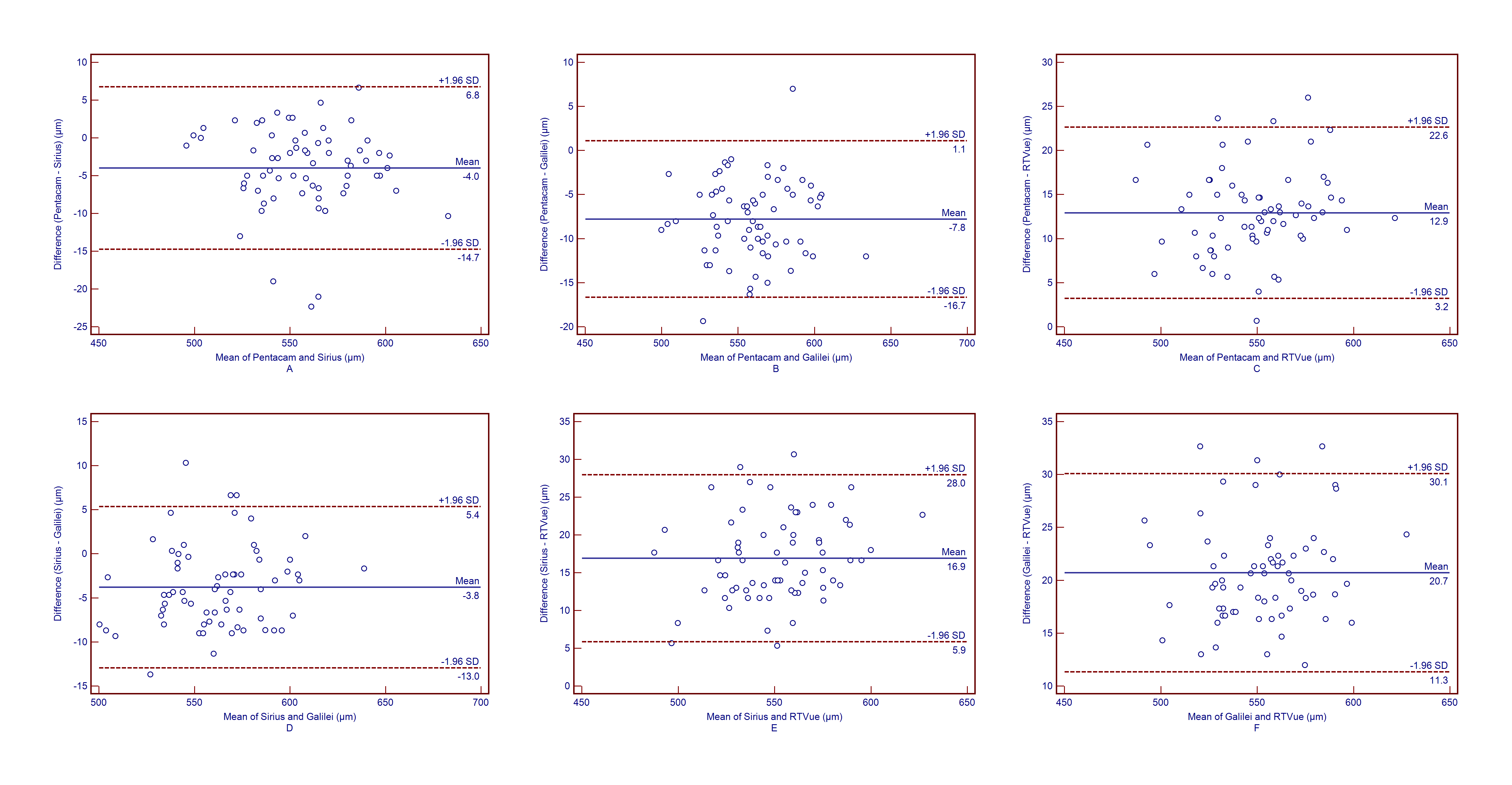

Supplement: Figure S3 — Bland-Altman plots of agreement in corneal thickness measurement of the superior location with a distant of 1 mm from the corneal apex (CTsuperior-2mm) among Pentacam, Sirius, Galilei, and RTVue OCT. The solid line indicates the mean difference (bias). The upper and lower lines represent the 95% LoA. (TIF) [file pone.0098316.s003.tif]

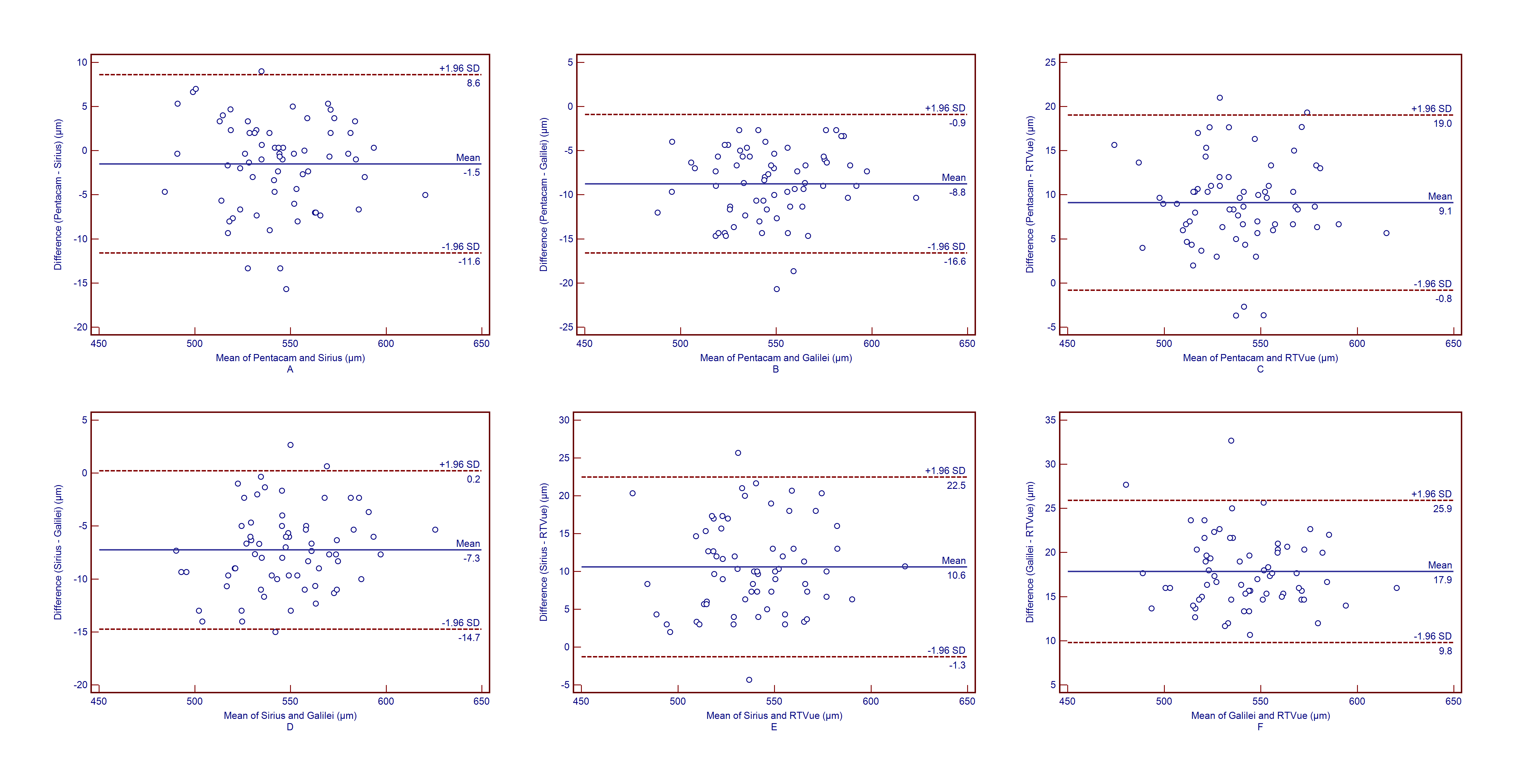

Supplement: Figure S4 — Bland-Altman plots of agreement in corneal thickness measurement of the inferior location with a distant of 1 mm from the corneal apex (CTinferior-2mm) among Pentacam, Sirius, Galilei, and RTVue OCT. The solid line indicates the mean difference (bias). The upper and lower lines represent the 95% LoA. (TIF) [file pone.0098316.s004.tif]

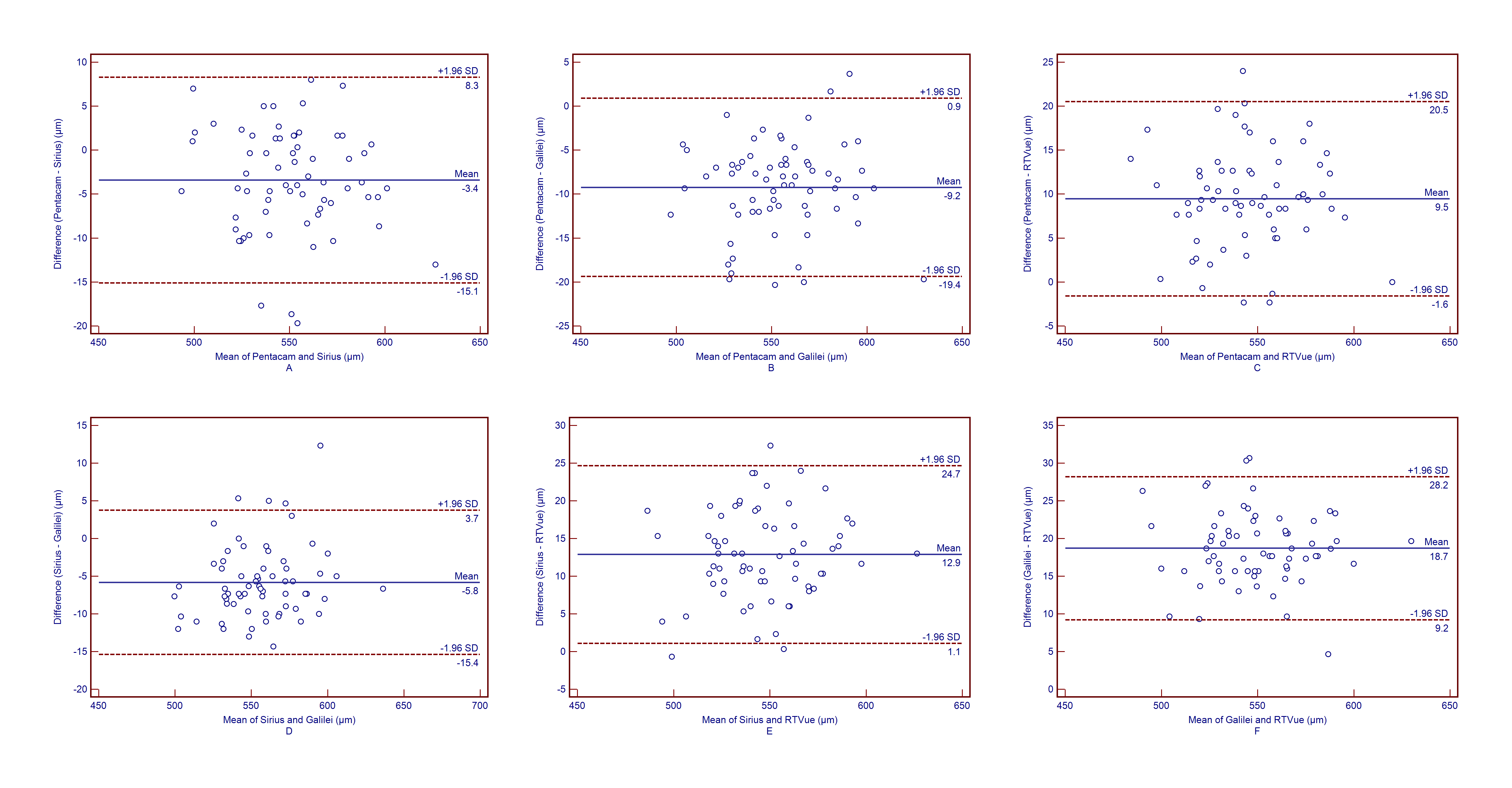

Supplement: Figure S5 — Bland-Altman plots of agreement in corneal thickness measurement of the nasal location with a distant of 1 mm from the corneal apex (CTnasal-2mm) among Pentacam, Sirius, Galilei, and RTVue OCT. The solid line indicates the mean difference (bias). The upper and lower lines represent the 95% LoA. (TIF) [file pone.0098316.s005.tif]

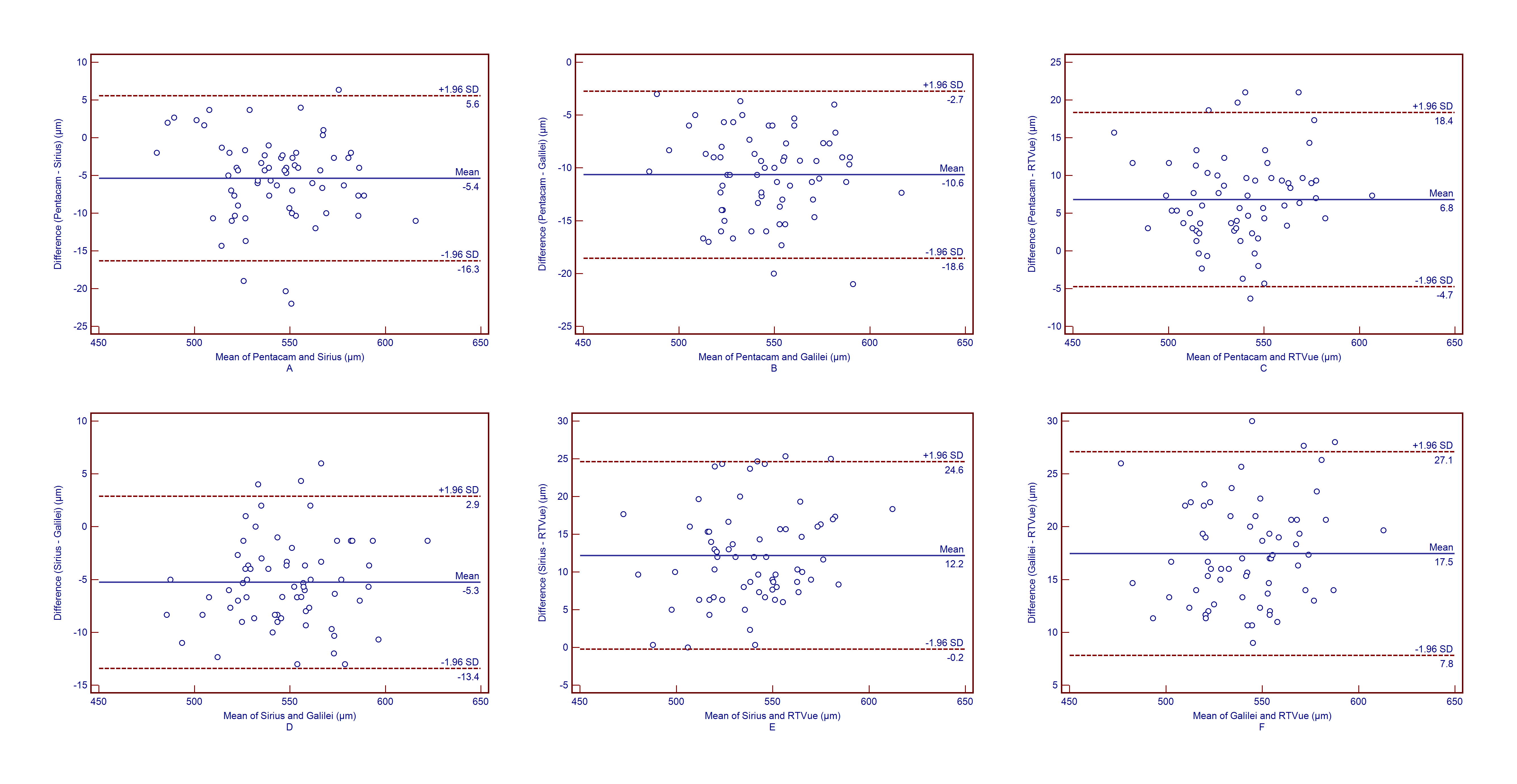

Supplement: Figure S6 — Bland-Altman plots of agreement in corneal thickness measurement of the temporal location with a distant of 1 mm from the corneal apex (CTtemporal-2mm) among Pentacam, Sirius, Galilei, and RTVue OCT. The solid line indicates the mean difference (bias). The upper and lower lines represent the 95% LoA. (TIF) [file pone.0098316.s006.tif]

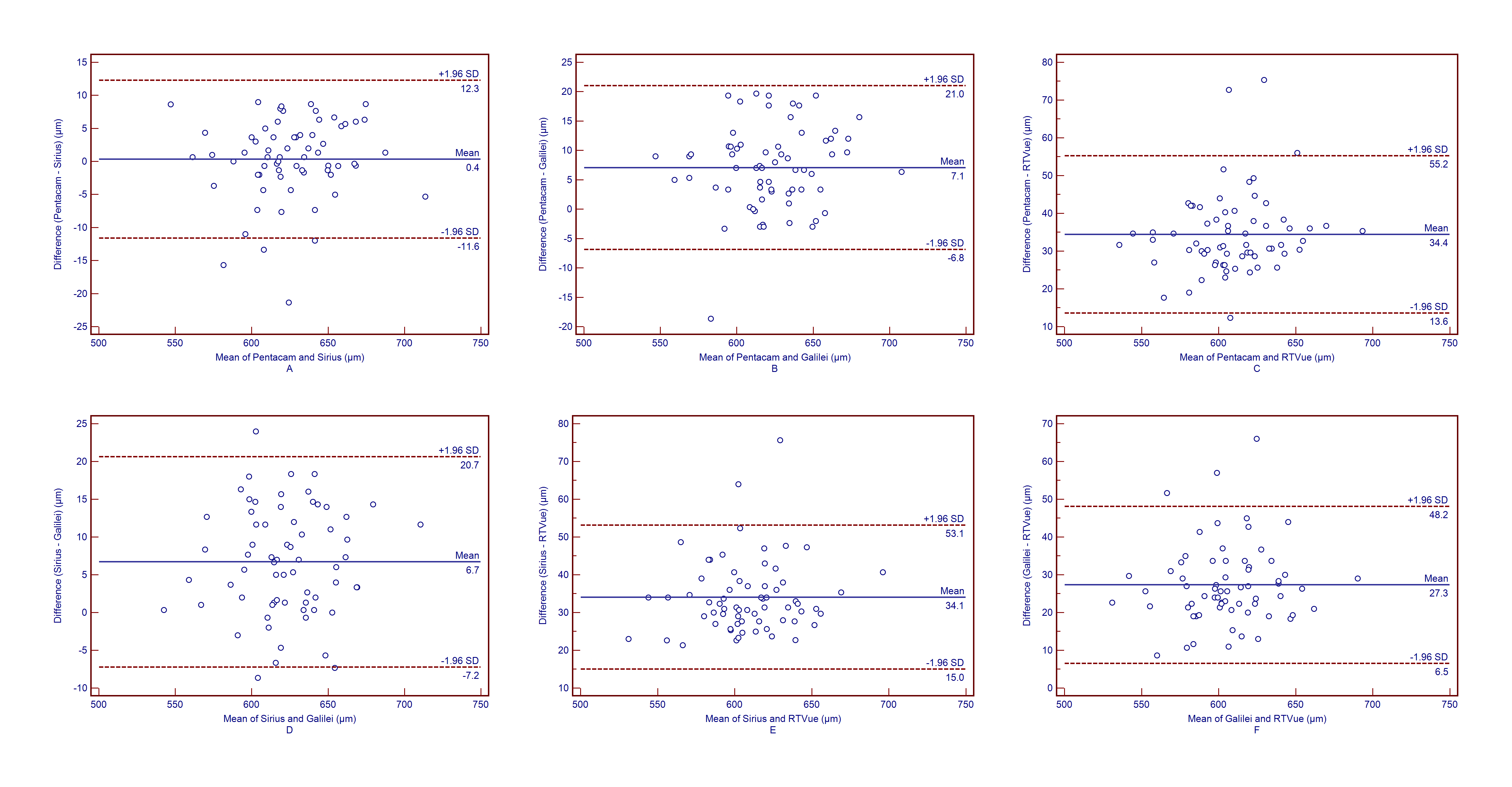

Supplement: Figure S7 — Bland-Altman plots of agreement in corneal thickness measurement of the superior location with a distant of 2.5 mm from the corneal apex (CTsuperior-5mm) among Pentacam, Sirius, Galilei, and RTVue OCT. The solid line indicates the mean difference (bias). The upper and lower lines represent the 95% LoA. (TIF) [file pone.0098316.s007.tif]

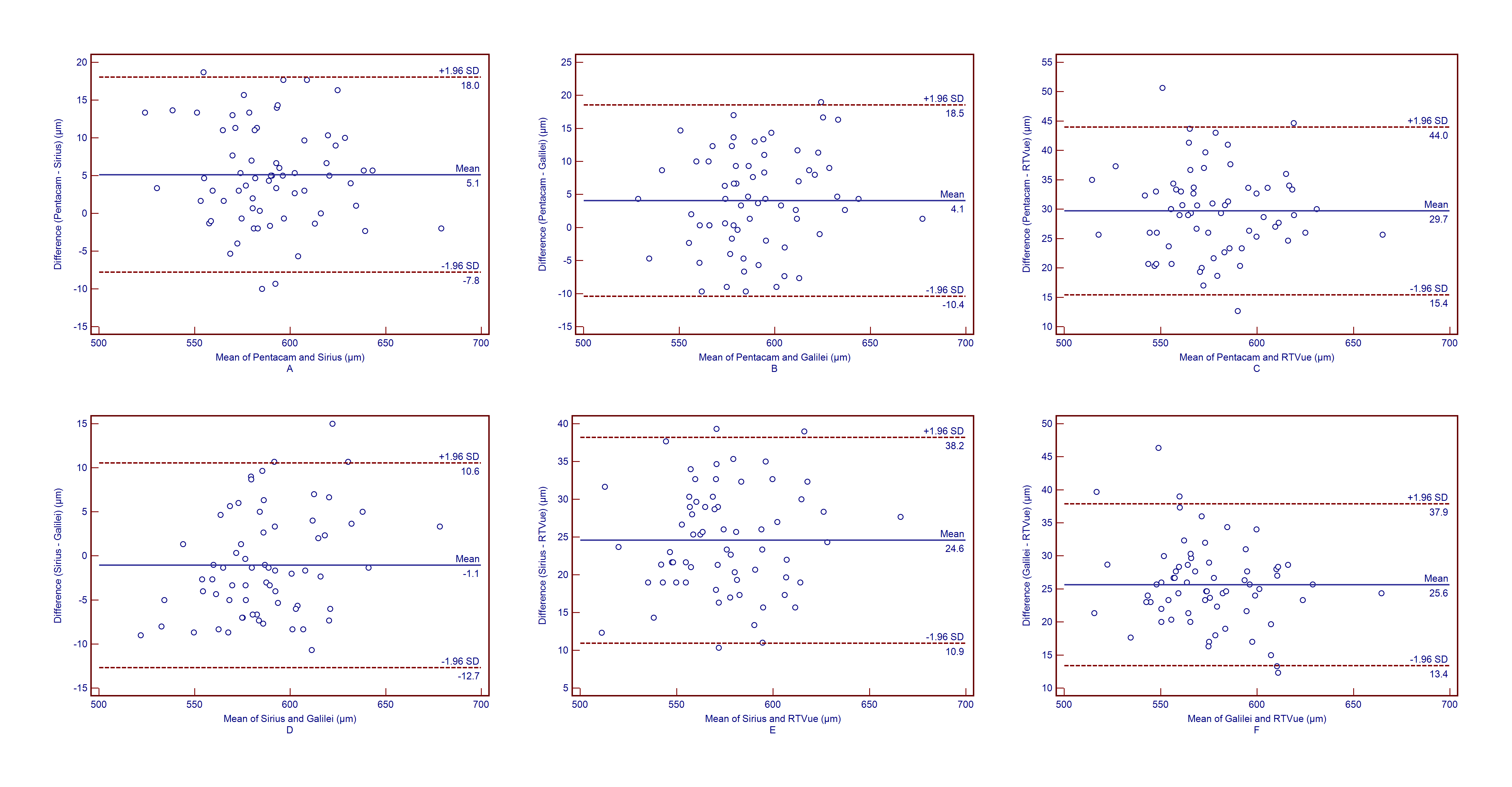

Supplement: Figure S8 — Bland-Altman plots of agreement in corneal thickness measurement of the inferior location with a distant of 2.5 mm from the corneal apex (CTinferior-5mm) among Pentacam, Sirius, Galilei, and RTVue OCT. The solid line indicates the mean difference (bias). The upper and lower lines represent the 95% LoA. (TIF) [file pone.0098316.s008.tif]

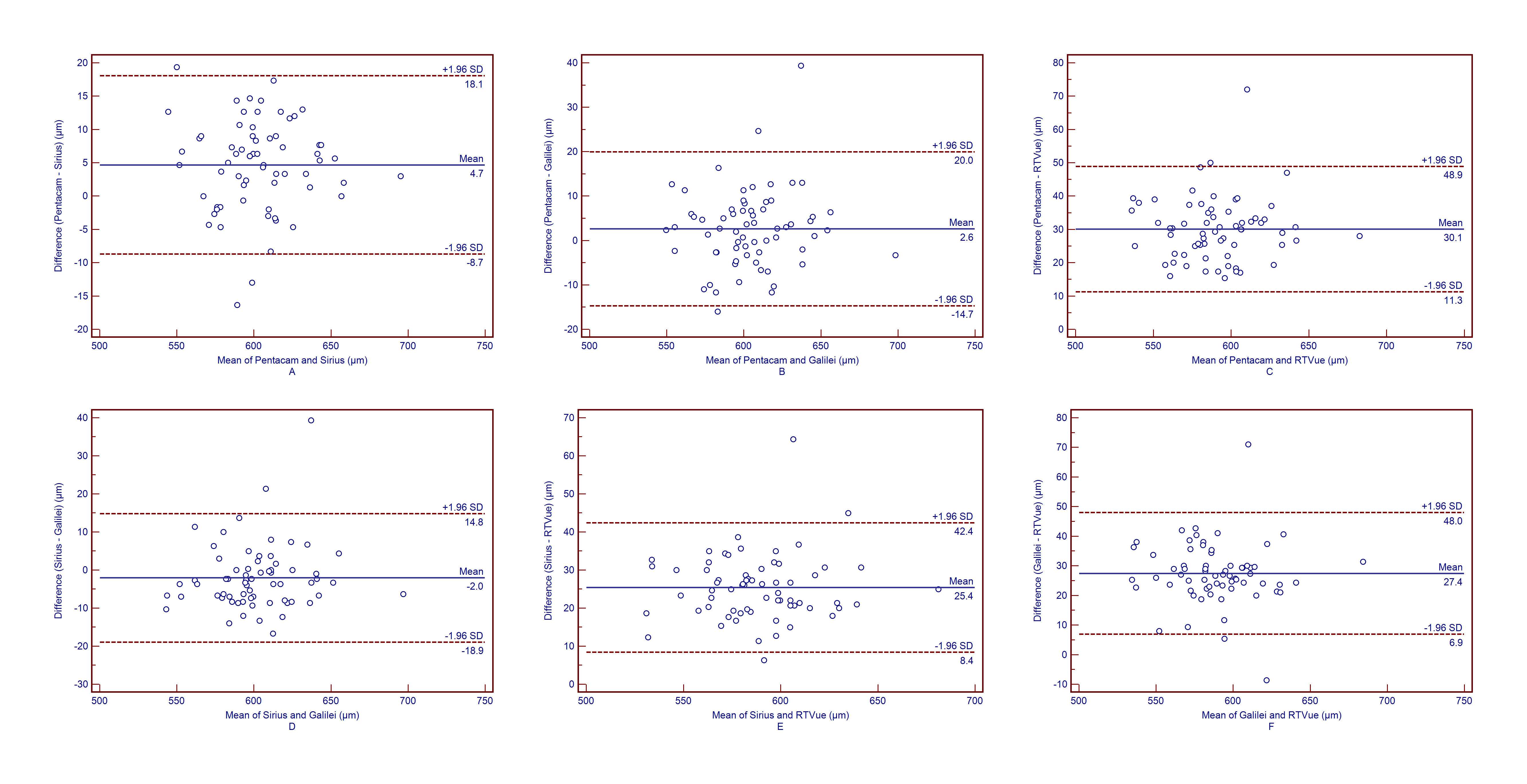

Supplement: Figure S9 — Bland-Altman plots of agreement in corneal thickness measurement of the nasal location with a distant of 2.5 mm from the corneal apex (CTnasal-5mm) among Pentacam, Sirius, Galilei, and RTVue OCT. The solid line indicates the mean difference (bias). The upper and lower lines represent the 95% LoA. (TIF) [file pone.0098316.s009.tif]

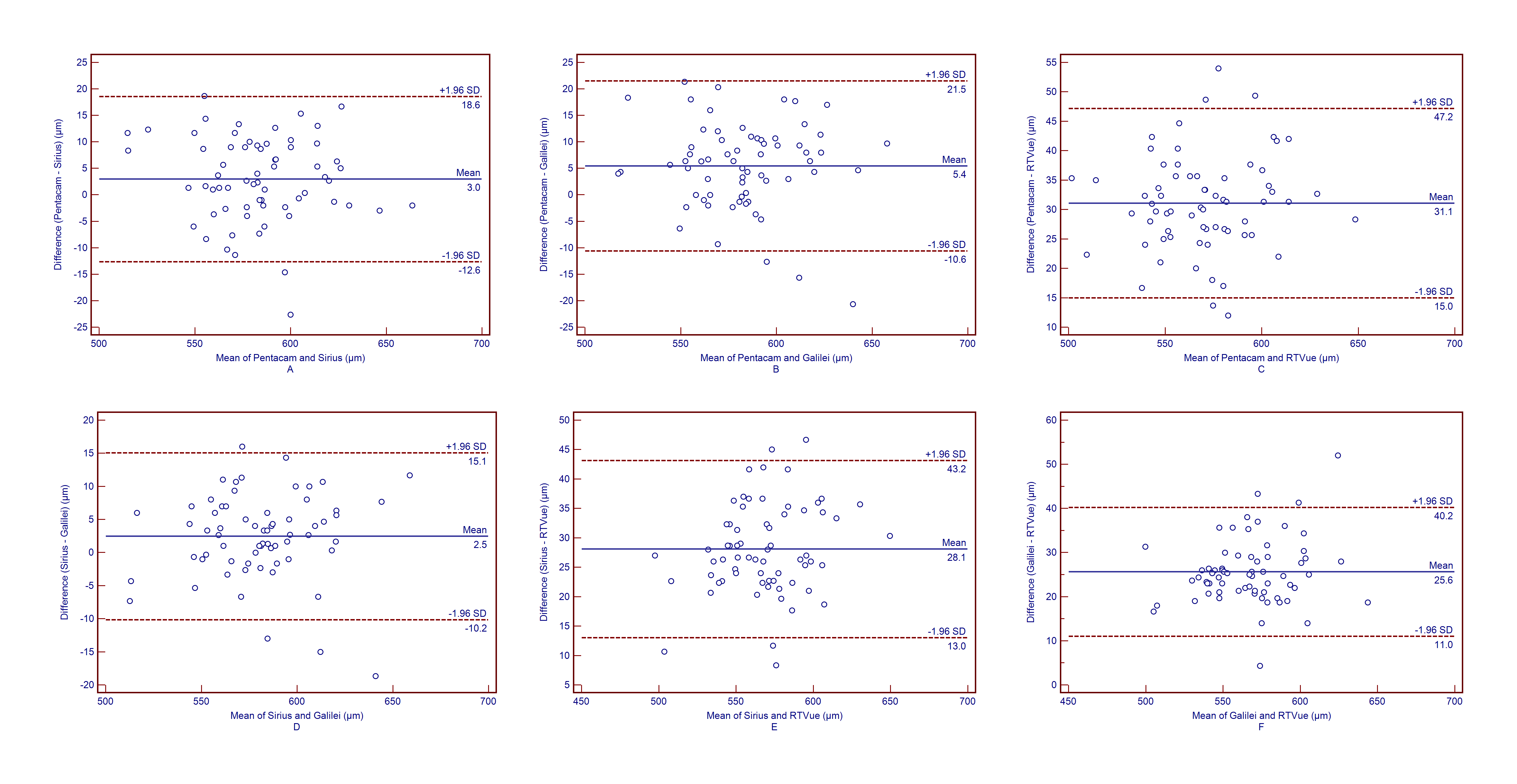

Supplement: Figure S10 — Bland-Altman plots of agreement in corneal thickness measurement of the temporal location with a distant of 2.5 mm from the corneal apex (CTtemporal-5mm) among Pentacam, Sirius, Galilei, and RTVue OCT. The solid line indicates the mean difference (bias). The upper and lower lines represent the 95% LoA. (TIF) [file pone.0098316.s010.tif]
