# Supplementary material for: Central and Midperipheral Corneal Thickness Measured with Scheimpflug Imaging and Optical Coherence Tomography
Source: PLoS One. 2014 May 22;9(5):e98316. doi: 10.1371/journal.pone.0098316 (PMC4031212; doi:10.1371/journal.pone.0098316)
Supplement: Table S1 — Mean difference of inferior 2 mm corneal thickness, corresponding results of Bonferroni post hoc comparison and 95% limits of agreement (LoA) among the 4 investigated devices. (DOCX) [file pone.0098316.s011.docx]

| Device Pairings | Mean Difference (μm) ± SD | *P* Value | 95% LoA (μm) |
| --- | --- | --- | --- |
| Pentacam - Sirius | -1.5 ± 5.2 | 0.129 | -11.6 to 8.6 |
| Pentacam - Galilei | -8.8 ± 4.0 | < 0.001 | -16.6 to -0.9 |
| Pentacam - RTVue | 9.1 ± 5.1 | < 0.001 | -0.8 to 19.0 |
| Sirius - Galilei | -7.3 ± 3.8 | < 0.001 | -14.7 to 0.2 |
| Sirius - RTVue | 10.6 ± 6.1 | < 0.001 | -1.3 to 22.5 |
| Galilei - RTVue | 17.9 ± 4.1 | < 0.001 | 9.8 to 25.9 |
| SD = Standard deviation. | | | |

Table S1. Mean difference of inferior 2mm corneal thickness, corresponding results of Bonferroni post hoc comparison and 95% limits of agreement (LoA) among the 4 investigated devices
