# Supplementary material for: Central and Midperipheral Corneal Thickness Measured with Scheimpflug Imaging and Optical Coherence Tomography
Source: PLoS One. 2014 May 22;9(5):e98316. doi: 10.1371/journal.pone.0098316 (PMC4031212; doi:10.1371/journal.pone.0098316)
Supplement: Table S2 — Mean difference of nasal 2 mm corneal thickness, corresponding results of Bonferroni post hoc comparison and 95% limits of agreement (LoA) among the 4 investigated devices. (DOCX) [file pone.0098316.s012.docx]

| Device Pairings | Mean Difference (μm) ± SD | *P* Value | 95% LoA (μm) |
| --- | --- | --- | --- |
| Pentacam - Sirius | -3.4 ± 6.0 | < 0.001 | -15.1 to 8.3 |
| Pentacam - Galilei | -9.2 ± 5.2 | < 0.001 | -19.4 to 0.9 |
| Pentacam - RTVue | 9.5 ± 5.6 | < 0.001 | -1.6 to 20.5 |
| Sirius - Galilei | -5.8 ± 4.9 | < 0.001 | -15.4 to 3.8 |
| Sirius - RTVue | 12.9 ± 6.0 | < 0.001 | 1.1 to 24.7 |
| Galilei - RTVue | 18.7 ± 4.8 | < 0.001 | 9.2 to 28.2 |
| SD = Standard deviation. | | | |

Table S2. Mean difference of nasal 2mm corneal thickness, corresponding results of Bonferroni post hoc comparison and 95% limits of agreement (LoA) among the 4 investigated devices
