# Supplementary material for: Central and Midperipheral Corneal Thickness Measured with Scheimpflug Imaging and Optical Coherence Tomography
Source: PLoS One. 2014 May 22;9(5):e98316. doi: 10.1371/journal.pone.0098316 (PMC4031212; doi:10.1371/journal.pone.0098316)
Supplement: Table S3 — Mean difference of temporal 2 mm corneal thickness, corresponding results of Bonferroni post hoc comparison and 95% limits of agreement (LoA) among the 4 investigated devices. (DOCX) [file pone.0098316.s013.docx]

| Device Pairings | Mean Difference (μm) ± SD | *P* Value | 95% LoA (μm) |
| --- | --- | --- | --- |
| Pentacam - Sirius | -5.4 ± 5.6 | < 0.001 | -16.3 to 5.6 |
| Pentacam - Galilei | -10.6 ± 4.0 | < 0.001 | -18.6 to -2.7 |
| Pentacam - RTVue | 6.8 ± 5.9 | < 0.001 | -4.7 to 18.4 |
| Sirius - Galilei | -5.3 ± 4.2 | < 0.001 | -13.4 to 2.9 |
| Sirius - RTVue | 12.2 ± 6.3 | < 0.001 | -0.2 to 24.6 |
| Galilei - RTVue | 17.5 ± 4.9 | < 0.001 | 7.8 to 27.1 |
| SD = Standard deviation. | | | |

Table S3. Mean difference of temporal 2mm corneal thickness, corresponding results of Bonferroni post hoc comparison and 95% limits of agreement (LoA) among the 4 investigated devices
