# Supplementary material for: Central and Midperipheral Corneal Thickness Measured with Scheimpflug Imaging and Optical Coherence Tomography
Source: PLoS One. 2014 May 22;9(5):e98316. doi: 10.1371/journal.pone.0098316 (PMC4031212; doi:10.1371/journal.pone.0098316)
Supplement: Table S4 — Mean difference of inferior 5 mm corneal thickness, corresponding results of Bonferroni post hoc comparison and 95% limits of agreement (LoA) among the 4 investigated devices. (DOCX) [file pone.0098316.s014.docx]

| Device Pairings | Mean Difference (μm) ± SD | *P* Value | 95% LoA (μm) |
| --- | --- | --- | --- |
| Pentacam - Sirius | 5.1 ± 6.6 | < 0.001 | -7.8 to 18.0 |
| Pentacam - Galilei | 4.1 ± 7.4 | < 0.001 | -10.4 to 18.6 |
| Pentacam - RTVue | 29.7 ± 7.3 | < 0.001 | 15.4 to 44.0 |
| Sirius - Galilei | -1.1 ± 5.9 | < 0.001 | -12.7 to 10.6 |
| Sirius - RTVue | 24.6 ± 7.0 | < 0.001 | 10.9 to 38.2 |
| Galilei - RTVue | 25.6 ± 6.3 | < 0.001 | 13.4 to 37.9 |
| SD = Standard deviation. | | | |

Table S4. Mean difference of inferior 5mm corneal thickness, corresponding results of Bonferroni post hoc comparison and 95% limits of agreement (LoA) among the 4 investigated devices
