# Supplementary material for: Central and Midperipheral Corneal Thickness Measured with Scheimpflug Imaging and Optical Coherence Tomography
Source: PLoS One. 2014 May 22;9(5):e98316. doi: 10.1371/journal.pone.0098316 (PMC4031212; doi:10.1371/journal.pone.0098316)
Supplement: Table S5 — Mean difference of nasal 5 mm corneal thickness, corresponding results of Bonferroni post hoc comparison and 95% limits of agreement (LoA) among the 4 investigated devices. (DOCX) [file pone.0098316.s015.docx]

|  | | | |
| --- | --- | --- | --- |
| Device Pairings | Mean Difference (μm) ± SD | *P* Value | 95% LoA (μm) |
| Pentacam - Sirius | 4.7 ± 6.8 | < 0.001 | -8.7 to 18.1 |
| Pentacam - Galilei | 2.6 ± 8.8 | 0.110 | -14.7 to 20.0 |
| Pentacam - RTVue | 30.1 ± 9.6 | < 0.001 | 11.3 to 48.9 |
| Sirius - Galilei | -2.1 ± 8.6 | 0.349 | -18.9 to 14.8 |
| Sirius - RTVue | 25.4 ± 8.7 | < 0.001 | 8.4 to 42.4 |
| Galilei - RTVue | 27.5 ± 10.5 | < 0.001 | 6.9 to 48.0 |
| SD = Standard deviation. | | | |

Table S5. Mean difference of nasal 5mm corneal thickness, corresponding results of Bonferroni post hoc comparison and 95% limits of agreement (LoA) among the 4 investigated devices
