# Supplementary material for: Central and Midperipheral Corneal Thickness Measured with Scheimpflug Imaging and Optical Coherence Tomography
Source: PLoS One. 2014 May 22;9(5):e98316. doi: 10.1371/journal.pone.0098316 (PMC4031212; doi:10.1371/journal.pone.0098316)
Supplement: Table S6 — Mean difference of temporal 5 mm corneal thickness, corresponding results of Bonferroni post hoc comparison and 95% limits of agreement (LoA) among the 4 investigated devices. (DOCX) [file pone.0098316.s016.docx]

| Device Pairings | Mean Difference (μm) ± SD | *P* Value | 95% LoA (μm) |
| --- | --- | --- | --- |
| Pentacam - Sirius | 3.0 ± 8.0 | 0.021 | -12.6 to 18.6 |
| Pentacam - Galilei | 5.4 ± 8.2 | < 0.001 | -10.6 to 21.5 |
| Pentacam - RTVue | 31.1 ± 8.2 | < 0.001 | 15.0 to 47.2 |
| Sirius - Galilei | 2.5 ± 6.4 | 0.018 | -10.2 to 15.1 |
| Sirius - RTVue | 28.1 ± 7.7 | < 0.001 | 13.0 to 43.2 |
| Galilei - RTVue | 25.6 ± 7.5 | < 0.001 | 11.0 to 40.2 |
| SD = Standard deviation. | | | |

Table S6. Mean difference of temporal 5mm corneal thickness, corresponding results of Bonferroni post hoc comparison and 95% limits of agreement (LoA) among the 4 investigated devices
